# Predicting climate change impacts on poikilotherms using physiologically guided species abundance models

**DOI:** 10.1073/pnas.2214199120

**Published:** 2023-04-03

**Authors:** Tyler Wagner, Erin M. Schliep, Joshua S. North, Holly Kundel, Christopher A. Custer, Jenna K. Ruzich, Gretchen J. A. Hansen

**Affiliations:** ^a^U.S. Geological Survey, Pennsylvania Cooperative Fish and Wildlife Research Unit, The Pennsylvania State University, University Park, PA 16802; ^b^Department of Statistics, North Carolina State University, Raleigh, NC 27695; ^c^Climate and Ecosystem Sciences Division, Lawrence Berkeley National Laboratory, Berkeley, CA 94720; ^d^Department of Fisheries, Wildlife, and Conservation Biology, University of Minnesota, St. Paul, MN 55108; ^e^Department of Ecosystem Science and Management, Pennsylvania Cooperative Fish and Wildlife Research Unit, The Pennsylvania State University, University Park, PA 16802

**Keywords:** cold-blooded, data fusion, extrapolation, freshwater fishes

## Abstract

Poikilothermic animals comprise most species on Earth and are especially sensitive to changes in environmental temperatures. Species conservation in a changing climate relies upon predictions of species responses to future conditions, yet predicting species responses to climate change when temperatures exceed the bounds of observed data is fraught with challenges. We present a physiologically guided abundance (PGA) model that combines observations of species abundance and environmental conditions with laboratory-derived data on the physiological response of poikilotherms to temperature to predict species geographical distributions and abundance in response to climate change. The model incorporates uncertainty in laboratory-derived thermal response curves and provides estimates of thermal habitat suitability and extinction probability based on site-specific conditions. We show that temperature-driven changes in distributions, local extinction, and abundance of cold, cool, and warm-adapted species vary substantially when physiological information is incorporated. Notably, cold-adapted species were predicted by the PGA model to be extirpated in 61% of locations that they currently inhabit, while extirpation was never predicted by a correlative niche model. Failure to account for species-specific physiological constraints could lead to unrealistic predictions under a warming climate, including underestimates of local extirpation for cold-adapted species near the edges of their climate niche space and overoptimistic predictions of warm-adapted species.

## Significance

Poikilotherms, often referred to as “cold-blooded” animals, are sensitive to changes in environmental temperatures. Climate change, therefore, represents a significant threat to this diverse group of animals. Predicting the effects of temperatures outside the range of previously observed conditions is difficult using observed data alone. We develop an approach to predicting climate change effects on poikilotherm abundance and distributions by combining laboratory-derived information about species’ temperature preferences and tolerances with field-based measurements of abundance. Using data from three fish species that differ in temperature preferences, we show that incorporating physiological information into model predictions yields more realistic predictions of local extirpation and changes in abundance under future climate scenarios.

Predicting species distributions and abundance under future climate scenarios is one of the foundations of climate science and adaptation. Predicting species responses to climate change is fraught with uncertainty, particularly when future conditions are predicted to exist outside the bounds of observed data (1). Most predictions of species responses to climate change are based on species occurrence and rely on field-derived data and correlative niche models (2). The use of presence only or presence/absence data—in contrast to models of abundance—is often driven by data availability, especially when modeling at large spatial extents or across many species and ecosystems. The reliance on correlative niche models—as opposed to mechanistic models—is largely because they do not require information about ecological processes structuring populations and they are relatively straightforward to implement (e.g., ref. 3). Both modeling approaches, however, have benefits and constraints. For example, mechanistic models are frequently based on fine-scale measurements of individual physiology and must be linked to population-level processes at coarser spatial and temporal scales (4). Furthermore, either approach may produce unrealistic predictions when extrapolated to future temperature conditions not experienced by animals in their current range (5, 6). For correlative niche models, predictions about species responses to future climates are often made by estimating climate-related regression parameters across the current range of observed conditions and then extrapolating these relationships to make predictions about responses to warming. This approach assumes that species-environment relationships are biologically meaningful and will continue under future temperatures that are often outside the observed range of temperature conditions (7). Such approaches rely on the realized rather than the fundamental niche of species and may have limited transferability when applied to new conditions (8). Statistical methods exist for reducing uncertainty associated with extrapolation, including nonrandom subsetting of data used for validation (1, 9). These approaches assume a continuation of the functional relationships between species and climate into unsampled space, when in reality, the relationship between temperature and poikilotherms frequently exhibits nonlinear or threshold dynamics that may not be estimated using field-derived data alone (10). Mechanistic models, in contrast, explicitly consider important processes that constrain demographic processes and species ranges and provide an ecological foundation for predicting responses to future climates (11). Mechanistic models, however, require information on processes that regulate populations that may not be readily available for many organisms (12) and may itself be uncertain. Given the potential limitations of either approach, hybrid models that combine correlative and mechanistic approaches offer an encouraging alternative (13).

Some hybrid approaches use “data fusion” to improve predictions of species responses to climate change (14, 15). For example, information on physiology, life history, and trophic interactions has been integrated to predict distributions of marine fishes under climate change scenarios (16). To date, climate change-motivated data fusion efforts have focused on modeling species presence or presence/absence. However, the tight link between climate and demographic processes suggests that changes in species abundance will either occur before or accompany range shifts for most organisms (e.g., ref. 17).

Understanding potential changes in species abundance, in addition to their distributions, has significant value for developing climate adaptation and management strategies. Managing species abundance is the focus of efforts related to threatened and endangered species, harvested species, and invasive species. To date, most efforts aimed at understanding shifts in species distributions in response to changing habitat conditions have been biased toward terrestrial ecosystems and birds and less than ∼3% of poikilothermic animals (18, 19). This bias toward terrestrial homeotherms leaves out poikilotherms, which comprise most species on Earth and represent a diverse group of organisms that are particularly sensitive to changes in thermal habitat conditions (20). Although there is a dearth of studies on the response of the distribution and abundance of poikilotherms to climate change, these taxa are well studied in terms of laboratory-derived physiological data on thermal performance and tolerance (21). Demographic processes and relative fitness of poikilotherms can be mechanistically linked to temperature preferences and thresholds, and these relationships can inform predicted responses to climate change (22). Because most species lack adequate data for developing mechanistic models of abundance, there is a need for data-fusion approaches that integrate field-derived data on species-environment relationships with mechanistic understanding of temperature controls on demographics in order to make predictions about abundance and distributions under a changing climate.

We propose a physiologically guided abundance (PGA) model for predicting the effects of temperature on the geographical distribution and abundance of poikilothermic animals. The PGA model fuses data from experimental studies with landscape-level monitoring of species abundance and environmental conditions. Most poikilothermic animals share a similar functional response in relative performance to changes in temperatures that can be derived from laboratory studies (23). This temperature–performance response curve is generalizable across diverse poikilothermic taxa and can be leveraged to guide predictions of abundance under a changing climate.

**Fig. 1.**
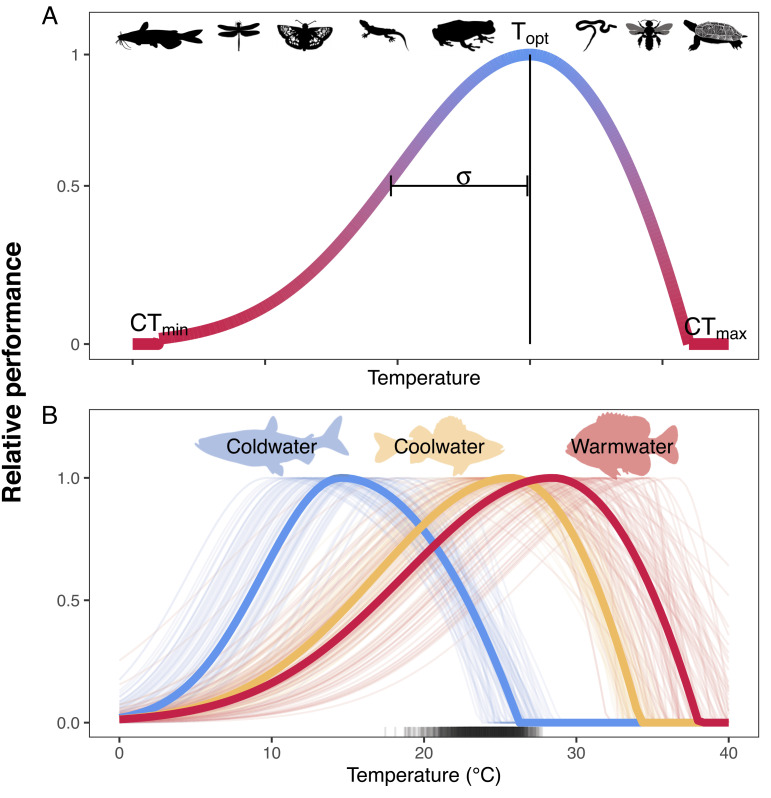
(*A*) Theoretical thermal performance curve for poikilothermic animals. The performance curve uses a Gaussian function to describe the ascending limb of the performance curve from zero at a critical minimum temperature (*CT*_*min*_) up to an optimal temperature (*T*_*opt*_) and a quadratic decline to zero at critical maximum temperature (*CT*_*max*_). The parameter *σ* is the scale parameter of the ascending Gaussian limb of the response curve from *CT*_*min*_ to *T*_*opt*_. (*B*) Performance curves for a cold-water (cisco), cool-water (yellow perch), and warm-water (bluegill) fish species derived from laboratory-based thermal tolerance studies. Thin lines represent uncertainty in thermal performance curves and are derived from 100 random draws of normally distributed thermal performance curve parameters, where the mean and SD of the normal distribution are based on literature-derived values. Thick lines represent the mean performance curve across the 100 draws. The rug plot shows the density of mean July water temperatures in Minnesota lakes. Silhouette images are from http://phylopic.org/ under creative commons license.

The performance response of poikilotherms to environmental temperature can be described by an asymmetrical concave curve, where relative performance—which could be quantified as changes in growth, reproductive rates, activity, or other metabolic or physiological changes—is maximized at an optimal temperature (*T*_*opt*_) and declines to zero at the minimum and maximum critical temperatures (*CT*_*min*_ and *CT*_*max*_, respectively; Fig. 1*A*). The asymmetry of the curve has important ramifications for species responses to climate change because increases in temperature beyond *T*_*opt*_ can quickly result in individuals reaching the heat-stressed zone of their tolerable thermal range. Failure to adequately capture the asymmetrical, descending limb of the thermal performance curve when predicting abundance and distributions under future climate scenarios may lead to biologically unrealistic predictions and is more likely to occur when making extrapolations beyond observed temperature ranges.

Temperature performance response curves and their relevant parameters are generally derived from laboratory and observational studies of individuals. Recent research has demonstrated a strong concordance between individual, lab-based physiological processes and population-level performance, and while the absolute value of thermal heating tolerances differed between lab and wild populations, both declined at similar rates with increasing temperatures (24). This temperature-performance link motivates our current work that fuses physiological response curves to inform predictions from species abundance models. Data fusion occurs by explicitly incorporating a thermal performance response curve into a statistical model of abundance. Information derived from experimental and observational studies is introduced into the analysis through informative prior distributions on model parameters describing the thermal performance response curve. This approach enables prediction of species abundance in response to changing thermal conditions even when temperatures exceed the bounds of observed conditions. Uncertainty in thermal response curves based on the range of literature values is also incorporated. We compare model predictions from the PGA model to an approach commonly used for predicting species distributions in a changing climate—a model that includes a linear and quadratic temperature effect, hereafter referred to as a naive model—for three freshwater fishes that differ in thermal preferences and tolerance across more than 1,300 lakes located in the Midwestern United States.

## Results

We evaluated model fit for the PGA and naive models using the Pareto smoothed importance-sampling leave-one-out cross-validation information criterion (PSIS-LOO-IC). The naive model provided a better fit to the observed data (*SI Appendix*, Table S1) indicating that the data-driven naive model produces more accurate predictions of distributions and abundance under current conditions. A naive model may also be useful for extrapolating under future climates if observed data span the entire potential thermal range of a species. However, because the observed temperature data for most species—including those used in our analysis—do not span the species entire tolerable temperature (or geographic) range (i.e., the data are truncated), extrapolation of relationships outside of these conditions using the naive model has the potential to produce unrealistic predictions. We argue that despite providing poorer fit to current data, by capturing this nonmonotonic relationship, the PGA model is likely preferred for extrapolating abundance and distributions of poikilotherms under future climate scenarios for species with truncated data and for species with truncated thermal ranges in nature, such as poikilotherms living near the equator.

**Fig. 2.**
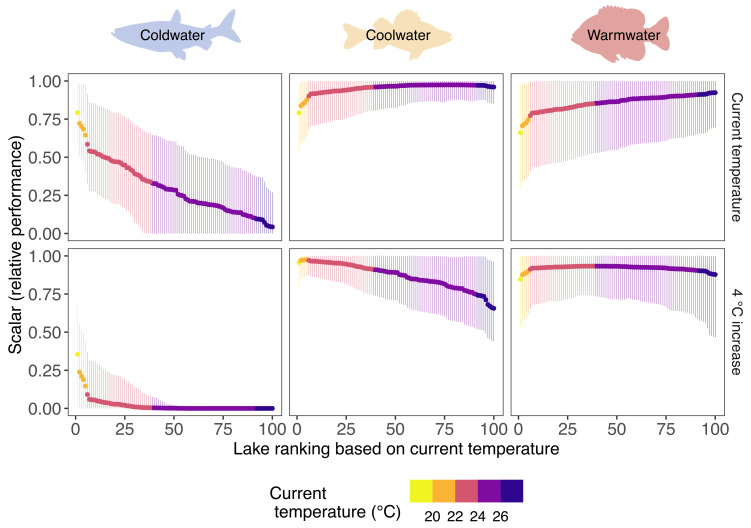
Posterior mean thermal performance scalars (solid circles) and associated 95% credible intervals (vertical lines) from the PGA model for cold-water (cisco), cool-water (yellow perch), and warm-water (bluegill) fish species for Minnesota lakes under current conditions (*Top* row) and a 4 °C increase in mean July water temperatures (bottom row). The figure shows 100 randomly selected lakes. Performance scalars range between 0 and 1, with 1 representing optimal performance and 0 representing extirpation. Silhouette images are from http://phylopic.org/ under creative commons license.

The predicted effects of warming (an increase of 1, 2, 3, or 4 °C increase in mean July water temperatures) on poikilothermic animals depended on a species’ thermal tolerance curve and degree of warming as well as the model used for prediction. Thermal habitat suitability and extinction probability are represented by the PGA model’s thermal performance scalar for each species. Even under current conditions and uncertainty in thermal performance curves, surface water temperatures of lakes exceeded the cold-water species’ thermal optimum, and performance rapidly approached zero with increasing water temperatures (Figs. 1*B* and 2). In contrast, current water temperatures are generally near the optimal temperature for the cool-water species, and their performance only slightly declined as water warmed. Current temperatures were generally slightly below the thermal optimum for the warm-water species, and therefore, warming conditions increased their performance as lake thermal habitat approached *T*_*opt*_ (Figs. 1*B* and 2).

### Extinction Probabilities of Cold-Adapted Species.

**Fig. 3.**
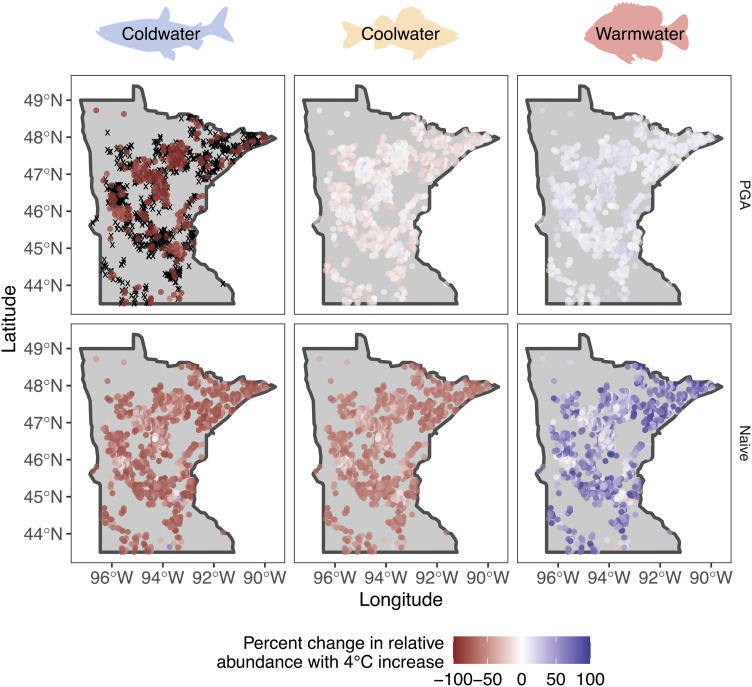
Predicted percent change in relative abundance for cold-water (cisco), cool-water (yellow perch), and warm-water (bluegill) fish species using the PGA (*Top* row) and naive (*Bottom* row) models for Minnesota lakes with a 4 °C mean July water temperature increase. Black × represents lakes with a predicted posterior probability of extinction > 90%. Silhouette images are from http://phylopic.org/ under creative commons license.

The PGA model predicted a posterior probability of extinction of > 90% for the cold-water species in some lakes across all levels of warming. At each level of warming, the temperatures of many lakes would exceed the critical thermal maximum of this cold-water species, given the uncertainty in thermal response curves, with the most stark differences at a 4 °C increase in mean July water temperature. Here, the PGA model predicted that 67% (n = 900 lakes) of lakes would be thermally unsuitable for the cold-water species, and they would be extirpated in 61% (n = 192) of the lakes they currently inhabit (Fig. 3). In contrast, the naive model cannot provide estimates of extinction probability and never predicted extirpation (abundance of 0) for the cold-water species even though mean July water temperatures exceeded *CT*_*max*_ in many lakes. The naive model did predict decreases in abundance for all lakes (Fig. 3) as the estimated quadratic effect captured the downward parabolic temperature effect for this species (*SI Appendix*, Table S1 and Fig. S1). However, the downward parabolic temperature effect fails to capture the asymmetry in thermal response to temperature and a biologically relevant *CT*_*max*_, and the cold-water species was predicted to persist even as temperatures exceeded *CT*_*max*_ by several degrees (Fig. 3).

### Predicting Abundance under Climate Change.

**Fig. 4.**
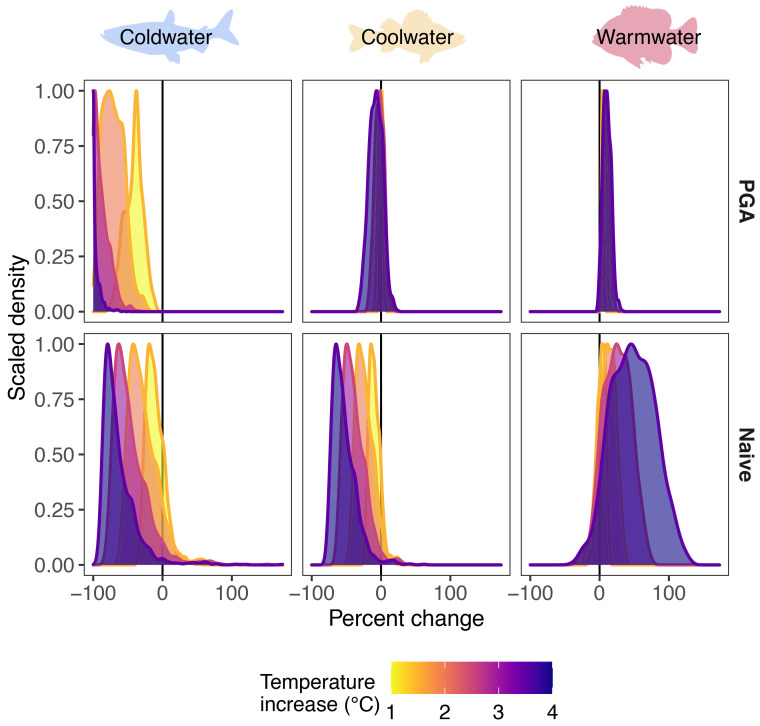
Predicted percent change in relative abundance for cold-water (cisco), cool-water (yellow perch), and warm-water (bluegill) fish species using the PGA (*Top* row) and naive (*Bottom* row) models for Minnesota lakes with a 1 to 4 °C increase in mean July water temperatures. Y-axis density estimate is scaled to a maximum of 1. Silhouette images are from http://phylopic.org/ under creative commons license.

Inferences about the effect of warming on the three thermal guilds were influenced by the portion of the thermal tolerance curve represented in the observed temperature data and the uncertainty in thermal response curves (Fig. 1*B*). Differences in abundance predictions between models are driven primarily by differences in functional responses to temperature. Species responses are dependent upon the range of observed temperatures relative to a species critical thermal maximum temperature and whether predicted temperatures approached or exceeded that critical maximum value. The effects of other environmental conditions on species abundance were remarkably consistent between the two modeling approaches (*SI Appendix*, Table S1). Model predictions between the two models diverged most notably when extrapolating outside the observed range of temperature data. For the cold-water species, the PGA model predicted a more rapid decline in abundance with increasing mean July water temperatures compared to the naive model. Severe declines in abundance of the cold-water species were predicted only by the naive model as temperatures warmed by 3 or 4 °C (Fig. 4), although both models predict extremely low abundance (naive model) or extinction (PGA model) at very warm water temperatures (e.g., > 30 °C; Fig. 4). For the cool and warm-water species, the PGA model predicted smaller and less variable percent changes in relative abundance in response to increasing water temperatures compared to the naive model (Figs. 3 and 4). However, differences in extrapolations of relative abundance under warming water conditions between the two models were in opposite directions for the cool and warm-water species. The PGA model predicted higher relative abundance with increasing water temperatures for the cool-water species and lower relative abundance for the warm-water species compared to the naive model.

Although the models often predicted no change (PGA model) or declines (naive model) in relative abundance for the cool-water species and increases in abundance for the warm-water species relative to current conditions, the purely data-driven naive model predictions were not informed by species physiological requirements, which led to unconstrained predictions under warming (Fig. 3 and *SI Appendix*, Figs. S2 and S3). For example, the cool-water species was predicted to decline by an average of 7% (range = −32% to 24%) with a 4 °C increase in mean July water temperature based on the PGA model but was predicted to decline by an average of 53% (range = −77% to 62%) based on the naive model. A similar pattern of more constrained predictions under the PGA model compared to the naive model was observed for the warm-water species, where the PGA model predicted a 10% average increase in abundance across all lakes (range = −2% to 34%) and the naive model predicted an average increase in relative abundance of 48% (range = −39% to 128%; Fig. 3).

## Discussion

Predicting the effects of climate change on species geographic distributions and abundance is a global priority for informing climate adaptation and mitigation strategies and biodiversity conservation (18, 25). However, the majority of species on Earth (and poikilotherms, in particular) lack field-derived abundance data to inform landscape-scale predictions, and even fewer have adequate physiological data available to enable mechanistic modeling of species responses to climate change. We develop and illustrate the utility of a physiologically guided abundance (PGA) model for predicting the effects of climate change on the geographic distribution and abundance of poikilothermic animals. In the absence of sufficient data to parameterize mechanistic models, the PGA model advances correlative niche-based modeling approaches by fusing temperature preference and tolerance data with species abundance data and environmental predictors of distributions and abundance.

Our approach relies on several assumptions when using a thermal performance function to inform climate change predictions. An underlying assumption is that there exists concordance between a species distribution and abundance and physiological performance—such that species will be more likely to be present and at higher abundance in locations that are physiologically optimal. However, there is the possibility that where a species occurs, or where peak abundance is observed, does not correspond to a physiologically optimum habitat. This mismatch between species distributions/abundance and demographic performance can result from factors that change performance over time (including management actions and competition with other species), which can bias predictions in response to climate change (26). We also assume a specific form of the thermal response function, but our approach is flexible and could be changed to accommodate species-specific variation in physiological responses to temperature, e.g., differences between eurytherms and stenotherms; (27).

Additional assumptions can be accommodated within the PGA modeling framework. For example, ontogenetic shifts in thermal tolerance are common among poikilotherms (28) and would be important to accommodate if suitable data were available (*SI Appendix*, Assumptions). We also assume that performance goes to zero at temperature values at or above *CT*_*max*_. This assumption could be relaxed, and performance could be set to any value deemed appropriate for a given objective e.g., 0.05 as in ref. 16. Furthermore, different values of *CT*_*max*_ or *T*_*opt*_ could be used for species that exist in different portions of their thermal range and may be acclimated to higher or lower temperatures. Consideration of the physiological end point(s) (i.e., traits) used to develop the thermal response curve is warranted to ensure that they are ecologically relevant and related to fitness (29). Other methodological differences in thermal performance experiments that may affect thermal performance values, e.g., acclimation temperatures; (30) could also be evaluated to help ensure comparability across studies. Lastly, the PGA model does not account for potential changes in thermal tolerance through plasticity or evolution; however, poikilotherms may have limited ability to change upper thermal limits in response to predicted temperature increases due to climate change (31). Our approach could be modified to incorporate additional variables relevant to a species abundance or persistent based on data availability and knowledge of life history.

Because many poikilothermic animals are physiologically adapted to specific thermal conditions (32), they represent some of the most important ecological indicators of climate change—they are the proverbial “canaries in the coalmine.” They also perform critical services to ecosystems, such as the provision of food and the pollination of agro-ecosystems and wild plant communities (33). Likewise, many poikilothermic animals are invasive outside their native ranges and have the potential to have climate-mediated catastrophic ecological impacts on biodiversity, social-ecological systems, and human health and well-being (34–37). Our method represents an approach to making predictions of poikilotherm distributions and abundance in a warming world.

## Materials and Methods

We illustrate the PGA model using three different freshwater fish species that differ in their distributions and thermal preference and tolerance (38). We contrast model predictions under different future climate scenarios among a cold-water stenotherm cisco *Coregonus artedi*; found in 24% [*n* = 316] of study lakes and widely distributed cool-water and warm-water species that are important for both commercial and recreational fisheries: yellow perch *Perca flavescens* found in 95% [*n* = 1274] of study lakes and bluegill *Lepomis macrochirus* found in 90% [n = 1206] of study lakes; Fig. 1*B*.

### Fish and Environmental Data.

Fish catch data were collected by the Minnesota Department of Natural Resources (MNDNR) using standard sampling methodology between 1998 and 2019 (39). We restricted our analysis to those samples collected between June 1 and September 30. All three species were sampled using gill nets and trap nets, commonly utilized gears for assessing the relative abundance of fishes in littoral (nearshore) and pelagic (off-shore) zones of inland lakes. Sampling effort consisted of one net (gill net or trap net) deployed for a 24-h sampling period.

Environmental predictor variables that are known to directly or indirectly influence inland lake fish abundance were included in the model. Environmental predictors were lake area, lake maximum depth, water clarity (Secchi disk depth), and the proportion of different land use and land cover in the lake watershed (*SI Appendix*, Table S2). Lake area and maximum depth were obtained from MNDNR public databases (https://gisdata.mn.gov/dataset/water-lake-basin-morphology). Water clarity was derived from remotely sensed Secchi disk depths (Max Gilnes, Rensselaer Polytechnic Institute, Troy, NY, United States, 05/2020, written communication). Lake water temperatures were simulated using a deep-learning model of daily water temperatures for lakes in the United States and are summarized here as mean July surface temperatures from ref. 40. Lake water temperature data and water clarity at the time of sampling were quantified using a 5-year rolling mean of annual values. The proportion of developed and agricultural land use and wetland land cover in each lake watershed was calculated based on the 2016 National Land Cover Database (41) and accessed through the LAGOSNE R package (42, 43).

### Climate Change Predictions.

We predict species distributions and abundance at each lake under current conditions and for a 1, 2, 3, and 4 °C increase in mean July water temperatures. A 4 °C increase corresponds to the predicted average regional increase in air temperature across the region for the 2071 to 2100 time period (44).

### Physiologically Guided Abundance Model.

Our model for catch data fits within the Poisson model framework for count data where the intensity is defined as the product of relative abundance (a measure of the size of a population that is assumed to be proportional to the true population abundance) and effort. The thermal performance curve is incorporated into the model for relative abundance, and the effort scaling captures both sampling effort and catchability.

Let *C*_*ijt*_ be the number of fish caught in lake *i* = 1, …, *I*, using sampling gear *j* = 1, …, *J* in year *t* = 1998, …, 2019 and *E*_*ijt*_ be the effort associated with each sample. We model the count data as

[1] $$\begin{array}{c} C_{\mathrm{ijt}}∼Pois\left({\tilde{E}}_{\mathrm{ijt}}\lambda_{\mathrm{it}}\right), \end{array}$$

where ${\tilde{E}}_{\mathrm{ijt}}$ = *E*_*ijt*_*θ*_*j*_, ***θ*** = [*θ*_1_, …, *θ*_*J*_]′ is the catchability vector, and *λ*_*it*_ is the relative abundance. We define

$$\begin{array}{c} \lambda_{\mathrm{it}}=P\left(T_{\mathrm{it}}\right)\exp\left(X_{\mathrm{it}}\beta \right), \end{array}$$

where **X**_*it*_ is a vector of lake covariates at the time of the sample, *β* is the coefficient vector, and *P*(*T*_*it*_) is the abundance scalar derived from the species-specific thermal performance function evaluated at temperature *T*_*it*_. The values of the function *P*(*T*_*it*_) range from 0 (poor performance if temperatures exceed *CT*_*max*_ or are below *CT*_*min*_) to 1 (optimal performance at *T*_*opt*_).

Although there are different thermal performance functions that can be used to describe the relationship between performance and temperature (45), we assumed an asymmetric thermal performance curve that uses a Gaussian function to describe the ascending limb of the performance curve up to *T*_*opt*_ and a quadratic decline to 0 at *CT*_*max*_ for the descending limb (46, 47). This performance curve has been previously used in studies on the effects of changing temperatures on poikilotherms (48, 49). We parameterize the thermal performance curve as

[2] $$\begin{array}{c} P\left(T\right)=\left\{\begin{array}{cc} exp(-(\frac{T-T_{\mathrm{opt}}}{2\sigma }{)}^{2}) & T\leq T_{\mathrm{opt}}\\ 1-(\frac{T-T_{\mathrm{opt}}}{T_{\mathrm{opt}}-CT_{\max}}{)}^{2} & T_{\mathrm{opt}}<T\leq CT_{\max}\\ 0 & T>CT_{\max}, \end{array}\right. \end{array}$$

where *σ* is the scale parameter for the Gaussian portion of the curve, and all other parameters are as described above and in Fig. 1. Although all parameters (*T*_*opt*_, *CT*_*max*_, and *σ*) can theoretically be estimated using abundance data (*SI Appendix*, Fig. S4), if sample locations do not span the entire temperature range of a species current distribution, then estimated parameters may be biologically inaccurate (e.g., an underestimated *CT*_*max*_), highly uncertain, or both. This is likely the case for many poikilotherms, where abundance data are available only within a portion of a species’ range.

### Accounting for Uncertainty in Thermal Response Curves.

The response metrics used for deriving thermal response parameters were most commonly growth rates (for *T*_*opt*_) and loss of equilibrium for *CT*_*min*_ and *CT*_*max*_. Using the growth of individuals to quantify *T*_*opt*_ is useful for fishes because it is related to survival, reproductive potential, life-span, and population dynamics (50–52). Loss of equilibrium is commonly used to assess thermal tolerance in fishes because it is assumed to represent death under wild conditions (53). However, the use of different response metrics results in uncertainty in thermal response curves (Fig. 1*B*). We incorporate this uncertainty in the model through the Bayesian framework by assigning prior distributions to these parameters based on the literature. Independent normal prior distributions are assigned to *T*_*opt*_ and *CT*_*max*_ using the literature-derived means and standard deviations reported in *SI Appendix*, Table S3. To fully specify the model, we assign prior distributions to all other model parameters, while *σ* is derived and assumed fixed (Section Species thermal tolerance data and *SI Appendix*, Table S3). The catchability vector, ***θ***, is modeled using a scaled Dirichlet prior with length *J* parameter vector *α* = [1, …, 1] and a scaling such that $\sum_{j=1}^{J}\theta_{j}=J$. For each coefficient parameter, we assign *β*_*l*_ ∼ *N*(0, 100) for *l* = 1, …, *r*, where *r* is the total number of estimated coefficients.

We use numerical integration to incorporate the uncertainty in *T*_*opt*_ and *CT*_*max*_ as an alternative to using the joint posterior of all parameters since the latter approach would be too heavily weighted by the likelihood given the data and thus would overwhelm the literature-derived values when using temperature data that do not span the entire range of values for which the temperature curves are estimated.

Specifically, we randomly sample *T*_*opt*_ and *CT*_*max*_ from their prior distributions, while ensuring that *T*_*opt*_ is less than *CT*_*max*_, providing a random realization of the thermal response curve. For each realization of *T*_*opt*_ and *CT*_*max*_, we fit the Bayesian model and obtain samples from the posterior distribution of all other model parameters with these values fixed. Numerical integration is obtained by repeating this process 100 times and aggregating the posterior distributions. This allows for the uncertainty in thermal response curves based on the literature to be propagated through to uncertainty in the other parameters and predictions.

### Species Thermal Tolerance Data.

Thermal tolerance data were compiled from the literature (54–63) and references therein. Because estimates of *σ* are not typically available in the literature, *σ* was derived using estimates of *CT*_*min*_ and *T*_*opt*_ following ref. 22, where *σ* = (*T*_*opt*_ - *CT*_*min*_)/4. If multiple *CT*_*min*_ values were available, the mean value, in addition to the mean *T*_*opt*_, was used when deriving the fixed *σ* for the thermal performance curve. If appropriate data were available, a prior could also be placed on *σ* (*SI Appendix*, Fig. S4).

All lake and landscape predictors were standardized to have mean zero and SD of one prior to analysis. Prior to standardization, because land use/cover variables and lake area and depth had heavily skewed distributions, they were logit-transformed and *log*_*e*_-transformed, respectively. For each model iteration, one for each of the 100 realizations of thermal response curves, a single Markov chain was run for a total of 3,000 iterations, of which the first 2,000 iterations were discarded as burn-in. Every 5^*th*^ sample was retained for a total of 200 samples per iteration, resulting in a grand total of 20,000 samples used to characterize the posterior distributions of the model parameters across all 100 iterations. Convergence was assessed visually through posterior distribution trace plots. Inference on the model parameters includes posterior mean estimates and 95% credible intervals. All models were fitted using the rstan package (64) called from the program R (65).

### Comparison of the PGA Model to the Naive Model.

We compared current and future climate predictions from the PGA model with predictions from a naive model that is purely data-driven and does not use thermal performance information. The naive model represents a common approach to modeling temperature effects on species distributions and abundance, where temperature is simply included in the linear predictor, in this case, as a quadratic term, i.e., *temperature*_*i*_ and *temperature*_*i*_^2^; (66) and (67). Specifically, the naive model is described by Eq. **1**, where the thermal performance scalar is removed and temperature and temperature^2^ are added as predictors in **X**_*it*_. Leave-one-out cross-validation (LOO) was performed to compare the PGA and naive model fits for each species (68). Using LOO, the log pointwise predictive density (ELPD) and the LOO information criterion (LOO-IC = -2 × ELPD) using Pareto smoothed importance sampling (PSIS) were calculated using the loo R package (69). Lower PSIS-LOO-IC values represent a model with a better fit to the data.

## Supplementary Material

Appendix 01 (PDF)

## Data Availability

The biological and environmental data associated with this manuscript are available on the Data Repository for University of Minnesota (GJAH is the point of contact): https://doi.org/10.13020/g1kt-4583. The code for Bayesian model fitting and posterior inference is available at https://doi.org/10.5066/P9YYGI5R.
