# Supplementary material for: Predicting climate change impacts on poikilotherms using physiologically guided species abundance models
Source: Proc Natl Acad Sci U S A. 2023 Apr 3;120(15):e2214199120. doi: 10.1073/pnas.2214199120 (PMC10104529; doi:10.1073/pnas.2214199120)
Supplement: Supplementary file 1 — Appendix 01 (PDF) [file pnas.2214199120.sapp.pdf]

Supporting Information for

Predicting climate change impacts on poikilotherms using physiologically guided species abundance models

Tyler Wagner, Erin M. Schliep, Joshua S. North, Holly Kundel, Christopher A. Custer, Jenna K. Ruzich and Gretchen J.A. Hansen

Tyler Wagner.
E-mail: twagner@usgs.gov

This PDF file includes:

Supporting text
Figs. S1 to S4
Tables S1 to S3
SI References

Supporting Information Text

Thermal performance curve assumptions. The use of a thermal performance curve and associated scalar in the PGA model explicitly assumes that information about a species' thermal performance is related to fitness and survival of wild populations (1). The strong concordance between individual, lab-based physiological heat tolerance and wild population-level performance across thermal gradients (2) suggests that this assumption is likely reasonable, and at a minimum provides a starting point for incorporating a mechanistic understanding of how poikilotherms may respond to a changing climate. This is especially useful for the majority of species that lack adequate data for process-based models.

Several additional assumptions have been identified when using thermal performance curves in the context of understanding climate change effects on poikilotherms (3). For example, the PGA model is unable to account for the potential effects of changes in the rate of temperature change or the duration and frequency of exposure to high temperatures on fitness, and therefore these factors are assumed to not be as important as temperature exposure at a given point in time relative to T_{opt} and CT_{max}. Although some of these assumptions cannot be directly addressed within the PGA modeling framework or are dependent on data availability. For example, the assumption that thermal performance curves will not change in the near-term is unknown, but is an implicit assumption of using thermal performance curves for assessing climate impacts. Other assumptions can be directly addressed within the modeling framework itself, given appropriate data. For our examples we assume that thermal performance does not change with developmental life-stage. This is certainly not the case for most species, as ontogenetic shifts in thermal tolerance is common among poikilotherms (4–6) and may vary seasonally (7). However, we lack sufficient data to relax this assumption in this study. If abundance and thermal performance data were collected for different life stages, the PGA model could be modified to allow for stage (or sex) -specific performance curves. Similarly, when modeling across large spatial extents, the assumption of a spatially-invariant thermal performance curve could be relaxed, if needed, and allowed to vary spatially. The PGA model is a flexible framework and easily modified to accommodate additional abundance, landscape predictor, and thermal performance data from observational and laboratory studies.

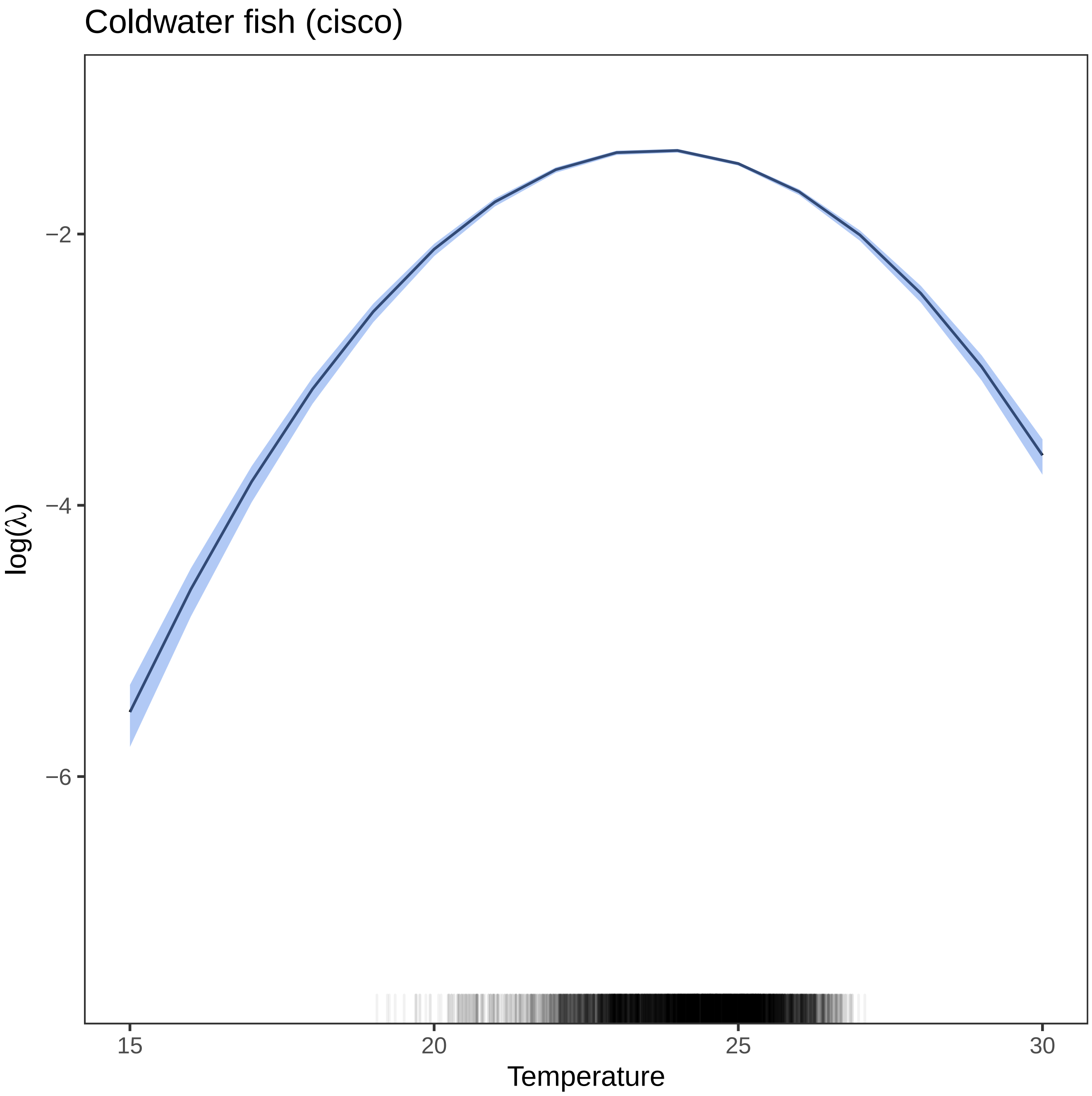

Fig. S1. Predicted relationship between mean July water temperature and log_e(relative abundance; λ) for a coldwater fish species from the naive model. Solid line is the posterior mean and shading represents 95% pointwise credible interval. Rug plot shows density of mean July water temperatures in Minnesota lakes.

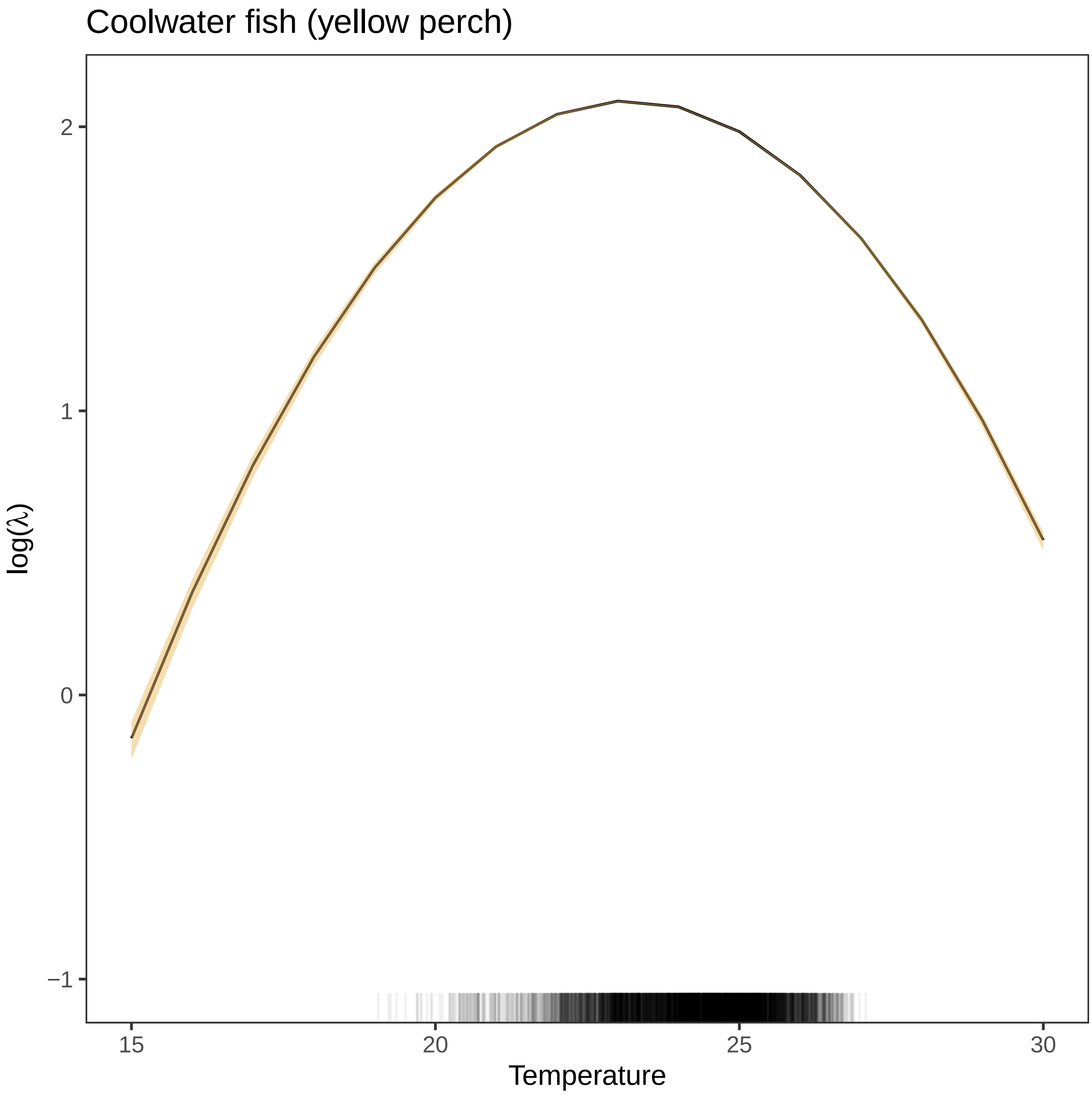

Fig. S2. Predicted relationship between mean July water temperature and log_e(relative abundance; λ) for a coolwater fish species from the naive model. Solid line is predicted posterior mean and shading is 95% pointwise credible interval. Rug plot shows density of mean July water temperatures in Minnesota lakes.

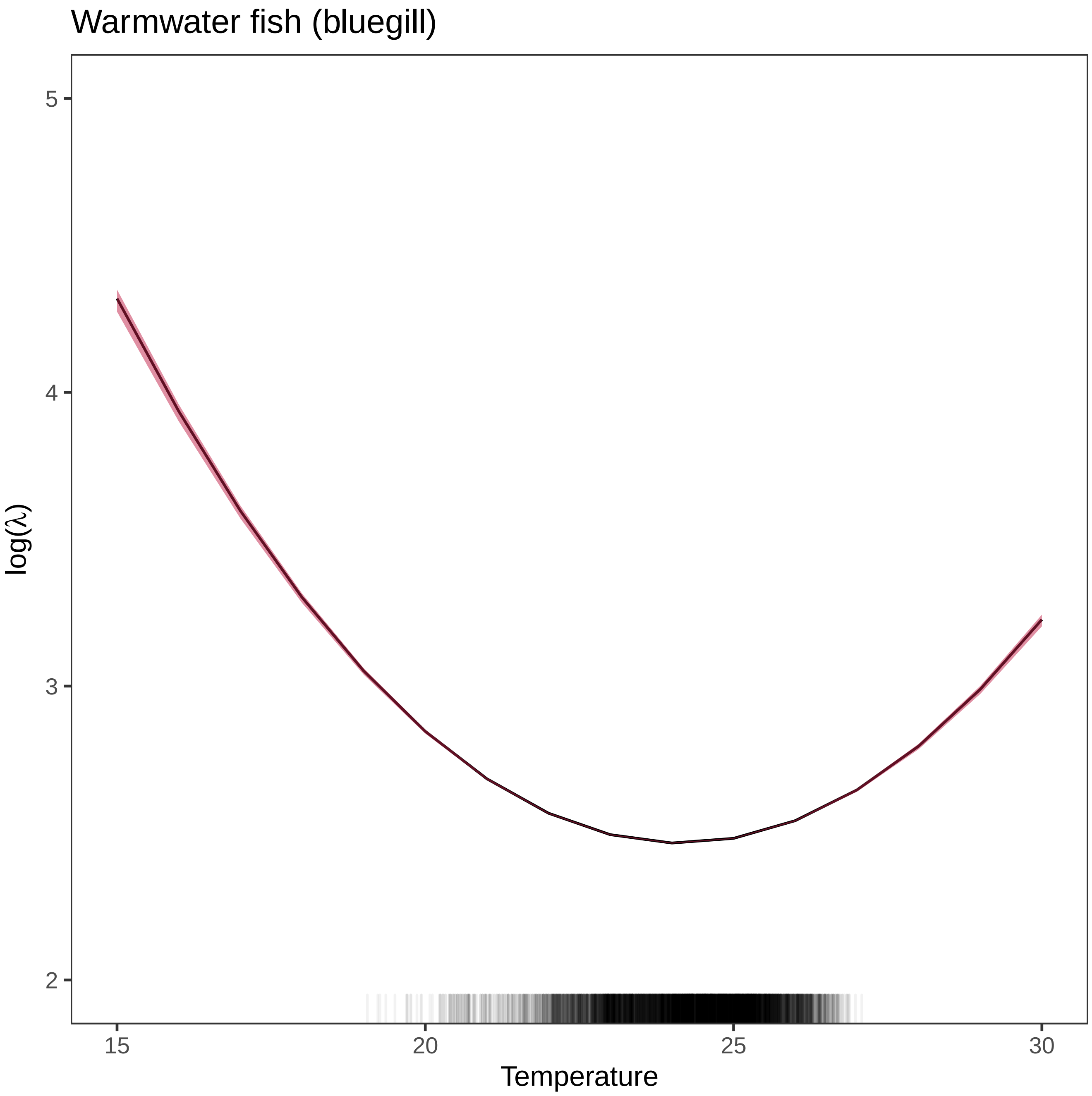

Fig. S3. Predicted relationship between mean July water temperature and log_e(relative abundance; λ) for a warmwater fish species from the naive model. Solid line is predicted posterior mean and shading is 95% pointwise credible interval. Rug plot shows density of mean July water temperatures in Minnesota lakes.

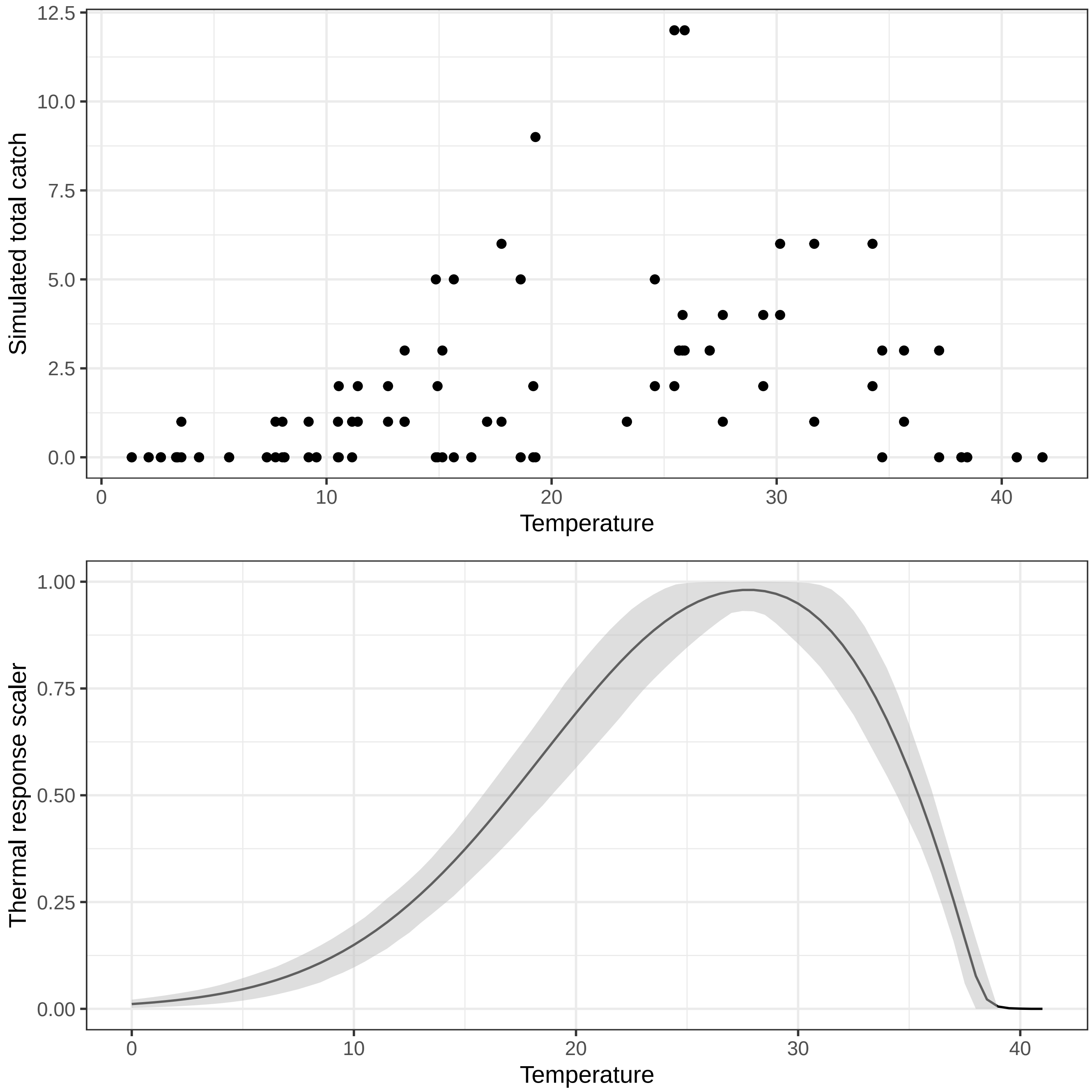

Fig. S4. Simulated catch data for 50 sites that span a theoretical species thermal range (top panel). Predicted thermal performance scalar for simulated abundance data where all parameters (CT_{max}, CT_{min}, and σ) are estimated from the data (bottom panel). Uniform priors were used for all parameters to bound biologically realistic values (see Online Code). Solid line is predicted posterior mean and shading is 95% pointwise credible interval.

Table S1. Parameter estimates (posterior means followed by 95% credible intervals in parentheses) describing the relationships between lake and watershed predictor variables on the abundance of a coldwater (cisco), coolwater (yellow perch), and warmwater (bluegill) fish species. Estimates are provided for the physiologically-guided abundance (PGA) model and naive model. The leave-one-out information criterion (PSIS-LOO-IC) is provided to compare models among species. Lower PSIS-LOO-IC values represent a model with a better fit to the data. Parameters θ_1 and θ_2 are catchability parameters, reported proportional to their sum, and temperature and temperature^2 are mean July water temperature and a quadratic mean July water temperature term in the naive model. NA = not applicable.

	PGA Model			Naive model		
Parameter	Cisco	Yellow perch	Bluegill	Cisco	Yellow perch	Bluegill
Intercept	-0.118 (-0.94,1.14)	2.06 (2.02,2.22)	2.68 (2.51,3.12)	-1.49 (-1.51,-1.47)	2.00 (1.99,2.01)	2.50 (2.50,2.51)
Developed	-0.123 (-0.243,-0.073)	-0.103 (-0.106,-0.1)	0.352 (0.348,0.355)	-0.04 (-0.053,-0.027)	-0.099 (-0.102,-0.096)	0.342 (0.341,0.344)
Agricultural	-0.471 (-0.512,-0.448)	-0.07 (-0.073,-0.066)	0.221 (0.207,0.236)	-0.463 (-0.477,-0.449)	-0.077 (-0.081,-0.074)	0.251 (0.248,0.253)
Wetlands	0.145 (0.118,0.189)	-0.041 (-0.044,-0.038)	-0.038 (-0.045,-0.031)	0.174 (0.16,0.188)	-0.046 (-0.049,-0.043)	-0.018 (-0.02,-0.016)
Secchi depth	0.137 (0.056,0.188)	-0.293 (-0.299,-0.288)	0.231 (0.228,0.234)	0.213 (0.198,0.228)	-0.299 (-0.302,-0.295)	0.235 (0.233,0.237)
Lake area	0.187 (0.161,0.252)	0.187 (0.184,0.19)	-0.209 (-0.211,-0.207)	0.166 (0.158,0.176)	0.192 (0.189,0.194)	-0.212 (-0.214,-0.21)
Lake depth	0.542 (0.311,0.657)	-0.023 (-0.033,-0.014)	0.169 (0.157,0.183)	0.73 (0.714,0.746)	-0.035 (-0.039,-0.032)	0.155 (0.153,0.158)
θ_1	1 (0.999,1)	0.909 (0.908,0.91)	0.187 (0.186,0.188)	1 (0.999,1)	0.909 (0.908,0.909)	0.187 (0.186,0.187)
θ_2	0 (0,0.001)	0.091 (0.09,0.092)	0.813 (0.812,0.814)	0 (0,0.001)	0.091 (0.091,0.092)	0.813 (0.813,0.814)
Temperature	NA	NA	NA	3.39 (3.13,3.63)	2.00 (1.93,2.07)	-1.38 (-1.42,-1.34)
Temperature^2	NA	NA	NA	-3.43 (-3.67,-3.16)	-2.06 (-2.13,-1.99)	1.37 (1.32,1.40)
PSIS-LOO-IC	621060.9	1171059.1	1901239.5	113571.6	1109927.9	1696105.7

Table S2. Summary statistics for environmental predictor variables used in models of fish abundance.

Predictor variable	Mean	Median	Minimum	Maximum
Lake area (ha)	889	355	15	39272
Lake maximum depth (m)	13	11	0.9	120
Water clarity (m)	2.7	2.8	0.4	5.9
Water temperature ($^\circ$C)	24.2	24.3	19.1	27.1
Urban land use (%)	4.2	1.0	0.0	76.1
Agricultural land use (%)	30.9	20.1	0.0	93
Wetland land cover (%)	16.8	11.6	0.0	95.3

Table S3. Literature-derived thermal performance curve parameter values used in the PGA model for a coldwater (cisco), coolwater (yellow perch), and warmwater (bluegill) fish species. The sample size (n) and standard deviation (sd) are in parentheses.Raw values are available in code available at https://doi.org/10.5066/P9YYGI5R. † The sd for Cisco CT_{max} was derived due to small sample size (n = 1). The derivation was done by calculating the ratio of the standard deviations for T_{opt} and CT_{max} for both yellow perch and bluegill (i.e., $T_{opt_{sd}}/CT_{max_{sd}}$). The mean of these two ratios was then multiplied by the Cisco T_{opt} sd to derive the CT_{max} sd.

Species	CT_{min}	T_{opt}	CT_{max}	σ
Cisco	0.3 (n = 1, sd = NA)	15 (n = 7, sd = 2.6)	26.2 (n = 1, sd = †1.29)	3.68
Yellow perch	1.1 (n = 1, sd = NA)	26 (n = 33, sd = 2.24)	34.5 (n = 3, sd = 0.81)	6.22
Bluegill	1 (n = 15, sd = 0.65)	28.3 (n = 44, sd = 4.2)	39.1 (n = 23, sd = 3.3)	6.83

References

1. V Kellermann, B van Heerwaarden, Terrestrial insects and climate change: adaptive responses in key traits. *Physiol. Entomol.* **44**, 99–115 (2019).
2. NL Payne, et al., Fish heating tolerance scales similarly across individual physiology and populations. *Commun. biology* **4**, 1–5 (2021).
3. BJ Sinclair, et al., Can we predict ectotherm responses to climate change using thermal performance curves and body temperatures? *Ecol. Lett.* **19**, 1372–1385 (2016).
4. XF Xu, X Ji, Ontogenetic shifts in thermal tolerance, selected body temperature and thermal dependence of food assimilation and locomotor performance in a lacertid lizard, eremias brenchleyi. *Comp. Biochem. Physiol. Part A: Mol. & Integr. Physiol.* **143**, 118–124 (2006).
5. Y Souchon, L Tissot, Synthesis of thermal tolerances of the common freshwater fish species in large western europe rivers. *Knowl. Manag. Aquatic Ecosyst.* p. 03 (2012).
6. NA Miller, AW Paganini, JH Stillman, Differential thermal tolerance and energetic trajectories during ontogeny in porcelain crabs, genus petrolisthes. *J. Therm. Biol.* **38**, 79–85 (2013).
7. AJ Turko, CB Nolan, S Balshine, GR Scott, TE Pitcher, Thermal tolerance depends on season, age and body condition in imperilled redside dace clinostomus elongatus. *Conserv. Physiol.* **8**, coaa062 (2020).
